# Predator Recognition in Rainbowfish, *Melanotaenia duboulayi*, Embryos

**DOI:** 10.1371/journal.pone.0076061

**Published:** 2013-10-16

**Authors:** Lois Jane Oulton, Vivian Haviland, Culum Brown

**Affiliations:** Department of Biological Sciences, Macquarie University, Sydney, Australia; The Australian National University, Australia

## Abstract

Exposure to olfactory cues during embryonic development can have long term impacts on birds and amphibians behaviour. Despite the vast literature on predator recognition and responses in fishes, few researchers have determined how fish embryos respond to predator cues. Here we exposed four-day-old rainbowfish (*Melanotaenia duboulayi*) embryos to cues emanating from a novel predator, a native predator and injured conspecifics. Their response was assessed by monitoring heart rate and hatch time. Results showed that embryos have an innate capacity to differentiate between cues as illustrated by faster heart rates relative to controls. The greatest increase in heart rate occurred in response to native predator odour. While we found no significant change in the time taken for eggs to hatch, all treatments experienced slight delays as expected if embryos are attempting to reduce exposure to larval predators.

## Introduction

Predation exerts one of the greatest selective pressures on prey organisms particularly during the vulnerable early juvenile growth phases [1]. In the presence of this selective force, it should not be surprising to discover that many organisms display innate anti-predator responses to visual or olfactory predator cues. In many circumstances, such innate responses are then finely honed following exposure to predators either directly (individual learning; [2]) or via the observation of attacks on conspecifics (social learning; [3]). In aquatic ecosystems the presence of predators is often signalled by chemosensory cues that may take a number of forms. In the simplest form, prey may be able to detect odours emanating directly from the predator. Some chemical cues, however, may indicate the threat of predation indirectly. Alarm substances released from damaged conspecifics, for example, can also signal that a predation event has taken place. Numerous papers have shown that the presence of such cues and their relative concentration, signal that a predator is in the vicinity, and the cues can be used to predict future predator attack [4], [5]. Consequently prey show anti-predator responses such as hiding or schooling when they detect these cues.

Innate responses to predator cues have been shown in a number of organisms. Even after 15 generations of isolation from predators, steelhead trout, *Oncorhychus mykiss*, still responded to the odour emanating from their natural predators [6]. Predator recognition may also be indicated by subtle observation of fish behaviour and or numerous neurophysiological variables associated with the flight or fight response. Both naïve Atlantic salmon, Salmo salar, and Nile tilapia, *Oreochromis niloticus*, increase opercular beat rates in the presence of predator cues [7], [8]. Similarly, heart rate is significantly elevated following predator detection [9]. Appropriate physiological responses to predatory cues that differ from exposure to control cues suggest that animals can differentiate between these cues and thus recognize them. Whether the recognition system is cognitive or an innate reflex is often difficult to determine. Where graded responses are illicited to cues that vary in threat content, however, it is likely that cognitive processes are involved as the animal refers to innate or learned templates during the recognition process [1].

It has been suggested that animals may be able to detect and respond to chemical cues during early embryonic stages. Salmonids, for example, may begin to imprint on the chemical signature of their home stream in the final stages of embryogenesis [10]. Chickens exposed to certain odours whilst still in the egg, later show preferences for such odours post-hatching [11]. Moreover, both salamander and frogs exposed to predator cues as embryos show appropriate anti-predator responses as tadpoles upon encountering the cues again [12].

Detection of predator cues by embryos can also effect the timing of hatching. Detection of potential egg predators speeds up development and causes early hatching in amphibians [13], while detection of potential larvae predators causes a delay in hatching [14], [15]. To date, however, few studies have examined predator detection by fish embryos despite the fact that the egg membrane is highly permeable [16] and early detection of predators may significantly enhance survival. Here we exposed four-day-old rainbowfish embryos (*Melanotaenia duboulayi*) to a host of predator cues and examined their response by observing changes in heart rate and hatch time.

## Methods

### Ethics Statement

Fish embryos are not covered by animal ethics legislation in Australia, but the adult stock and the entire protocol was approved by the Macquarie University Animal Ethics Committee (ARA 2011/024).

### Brood stock and culture

*M.duboulayi* eggs were obtained from brood stock originating from a wild population captured at Wilsons River near Lismore NSW in 1989. 14 adult fish were maintained in an isolated, 110 L glass aquarium containing aged tap water. The aquarium was furnished with river gravel and a filter. Temperature was maintained at 26±1°C and photoperiod kept constant on a 10 h light: 14 h dark cycle.

Two days prior to spawning, fish were fed to satiation with commercial flake fish food twice daily supplemented with 150 ml of thawed bloodworms at midday. Six sterilized spawning mops, consisting of bundles of green acrylic 8 ply thread suspended in the water column with polystyrene floats, were placed into the broodstock tank. Mops remained in place for 48 h during which time spawning occurred.

Following egg deposition, the mops were removed, treated in a Methylene blue solution (0.25 ml L^-1^) for 30 s to minimize fungal infections and transferred to isolated egg incubating chambers (20×38×20 cm). The aged water in the chambers was aerated to enhance oxygenation. Four-days post fertilization, individual eggs were gently teased from the mops and their hear rate monitored as outlined below. This time-point was chosen as heart chamber development and blood pigmentation in a closely related species (*M. fluviatilis*) is readily observed at this stage of development [17].

### Test Water

Tests were conducted in petri dishes containing 14 ml of synthetic water (hardness: 80 to 90 mg CaCO_3_ L^-1^), which was prepared in the laboratory according to Marking & Dawson [18]. Each litre of water contained: 96 mg of NaHCO_3_, 130 mg of MgSO_4_.7H_2_O, 4 mg KCL, and 60 mg CaSO_4_.2H_2_O dissolved in Milli-Q water (Millipore, USA) using a stirrer bar. The pH adjusted to 7.5 with 0.1 M HCL, vacuum filtered through a 0.45 µm pore membrane and stored in the dark at 4°C prior to use.

### Stimulus preparation

Stimulus preparation was based on that outlined elsewhere [19]. Briefly, a single spangled perch (120 mm standard length (SL)) and goldfish (140 mm SL) were established in 110 L aquaria and the filters turned off for 24hrs. Scented water was then extracted and frozen (−20°C) in 1 ml aliquots. Conspecific extract was created by killing an adult rainbowfish by decapitation and immediately removing the skin (1 cm^2^). The skin was placed on ice, crushed in 1 ml of synthetic water and passed through filter paper (6 µm, Advantec). The final solution was increased to 10 ml using synthetic water and stored in 1 ml aliquots in a freezer. Both the rainbowfish and the goldfish had been fed commercial flake food (Tetramin for tropical fish) while the spangled perch were fed frozen prawns supplemented with flake. Thus the diet of the fish was unlikely to influence the behaviour of the embryos.

### Experimental protocol

Harvested eggs were placed in 10 ml of synthetic water in small plastic petri-dishes. Eggs were examined in batches of five and assigned to one of four chemical cue treatments: 1) control, 32 µl of synthetic water; 2) Conspecific extract, 32 µl of rainbowfish odour; 3) Predator, 32 µl of spangled perch (*Leiopotherapon unicolor*) odour; 4) Novel predator, 32 µl of goldfish (*Carassius auratus auratus*) odour. The odour introduced to each petri dish was psuedorandomised to control for time of day and stress induced by repeatedly harvesting eggs.

The time taken for 100 heartbeats to occur in each egg was observed using a dissection microscope at 40× magnification (see Movie S1). Observation of each egg was repeated and the two counts averaged and converted to beats min^-1^. A total of 20 eggs were examined for each treatment. Data were normally distributed and analysed using ANCOVA with treatment as the fixed effect, petri-dish number as a covariate and heart rate as the dependent variable.

Following observations of heart rate, the eggs were left in their solutions so that hatch time could be recorded. In addition, a further sample of 20 eggs per treatment were harvested and placed directly into petri dishes to determine if our observations induced changes in hatch time and hatching success due to handling stress. The incidence of hatching was recorded daily until all embryos could be recorded as either hatched or dead. Hatch data was analysed using ANOVA.

## Results

**Figure 1:**
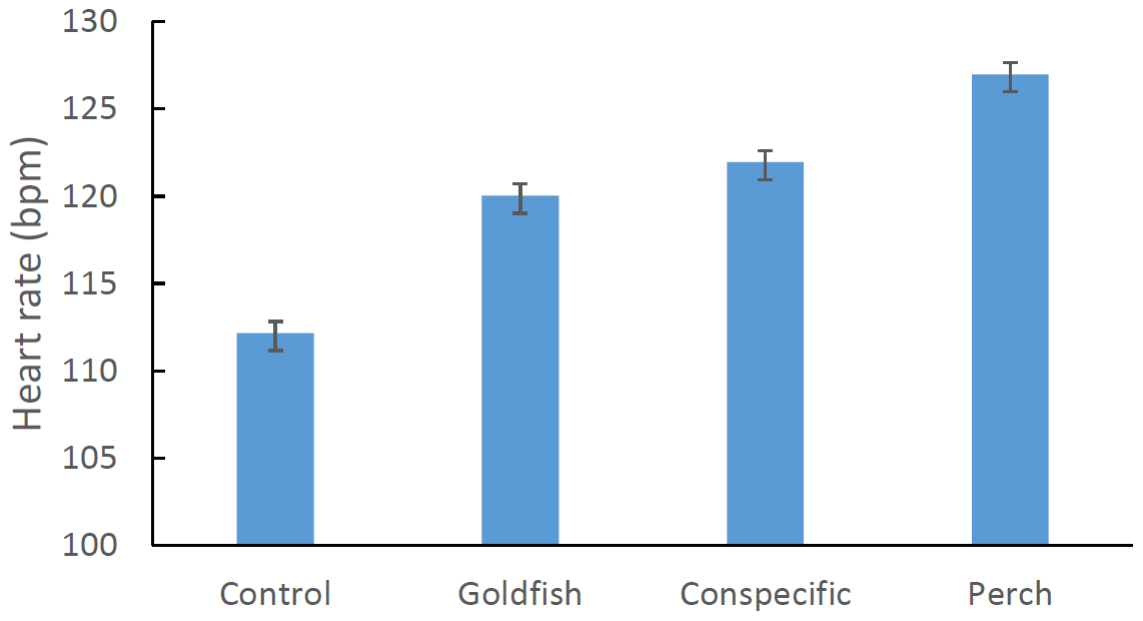
Mean (±SE) heart rate (beats per minute) of rainbowfish embryos exposed to a range of chemical cues. All cues induced a significant increase in heart rate relative to the control (distilled water).

Analysis of the heart-rate data showed a highly significant effect of cue (ANCOVA: F_3, 75_ = 14.989, P<0.001; Fig. 1). Post-hoc analysis revealed significant differences between all treatments (Fisher's PLSD: P<0.03 in all cases) with the exception of conspecific extract and goldfish odour (Fisher's PLSD: P>0.05). All odours elicited a faster embryonic heart rate relative to the control (synthetic water), with the native predator (silver perch) odour producing the greatest increase in heart rate. Heart rate significantly increased with petri-dish number indicating that the eggs became increasingly stressed as we repeatedly sampled different eggs from the mops over the course of the day (ANCOVA: F_1, 75_ = 20.548, P<0.001).

Examination of the hatch time data showed no differences between cues (ANOVA: F_3, 84_ = 0.592, P = 0.622) nor did handling influence hatching time (ANOVA: F_1, 84_ = 0.084, P = 0.773). In general, however, eggs exposed to predator cues tended to hatch slightly later than controls. Post-hoc analysis using a one-tailed t-tests based on the assumption that embryos should delay hatching when detecting larval predators suggested that the hatch date of eggs exposed to conspecific extract showed a marginally delayed hatching relative to the control eggs (t = 1.556, P = 0.063) and while both the other treatments showed similar trends they were not significant (goldfish: t = 1.302, P = 0.099; perch: t = 0.85, P = 0.199). Hatching success did not differ between treatments (ANOVA: F_3, 24_ = 0.929, P = 0.442; average 42.5%), however those eggs that were handled were less likely to hatch than those that were not (ANOVA: F_1, 24_ = 13.636, P = 0.001).

## Discussion

Rainbowfish embryos can distinguish between chemical cues emanating from various potential predators and from alarms substances released from damage inflicted on conspecifics. While a substantial increase in heart rate was observed in response to a novel predator (goldfish) relative to control levels, the greatest response was to the native predator (spangled perch). The response to the conspecific extract was indistinguishable to that of the goldfish. Quite clearly these embryos have had no prior exposure to predators given that they were raised in isolated aquaria thus the recognition system must be entirely innate. What is more surprising is the fish that the eggs were derived from have been in captivity for multiple generations [20]. Similar observations have been made in juvenile steelhead trout that have been isolated from predators for 15 generations [6]. While previous comparative studies of the anti-predator behaviour of rainbowfish have shown that isolation from predators over geological time scales can result in naiveté [21], [22], evidently innate predator recognition systems can be relatively long lived even in the absence of direct selection.

While we observed no significant shift in hatch day in response to predator cues, there was a tendency for all treatments to delay hatching relative to controls. The difficulty we face, however, is that hatching success is relatively low (ca 42%) and the embryonic stage is very short (just 7 days at 26°C), thus in order to detect significant delays in hatch date we undoubtedly require more power. Such low hatching success is typical of rainbowfish where individual females spawn hundreds of eggs per week, but this was further exacerbated by handling the eggs during the experiment. Most of them succumb to fungal infections in the lab but the hatch rate in the wild is likely to be significantly lower. It is interesting to note, however, that the eggs tended to delay hatching after exposure to the predator cues which is what would be expected if the cues were emanating from larval rather than egg predators [13], [14], [15]. Larval rainbowfish are only 4–5 mm long when they hatch and they undoubtedly fall prey to a wide range of predators including small fish and invertebrates. Juvenile spangled perch and goldfish are both well known for their broad dietary niche and both attack and consume larval fishes when they encounter them.

While we have clearly shown that rainbowfish larvae can detect and differentiate between predator cues, it remains unknown what the longer-term effects of this early exposure might be. Research in amphibians suggest that exposure to predator cues during embryogenesis can lead to appropriate avoidance behaviour as larvae [12]. No such response was observed in Atlantic salmon fry when exposed to pike odours between 27 and 1 day pre-hatch [23]. However, there are bound to be a number of other physiological and behavioural costs associated with accelerating or decelerating development. Not least of which is the potential for developmental instability. Clearly heart rate increases in fish embryos during exposure to predator cues, perhaps an indication of underlying stress, which may affect hatching success and larval behaviour. Studies on Atalantic salmon have shown that maternal stress has great impact on key larval characteristics including reduced body size, yolk sac volume, and an increase in morphological malformations [24]. Future studies will need to pay close attention to these potential long-term impacts of predator exposure during embryogenesis.

## Supporting Information

Movie S1 **A movie showing the heart beat and circulation of a rainbowfish embryo.**
(AVI)
